# P-1403. Inpatient Interferon Gamma Release Assays: Novel Tool or Alluring Pitfall

**DOI:** 10.1093/ofid/ofaf695.1590

**Published:** 2026-01-11

**Authors:** Rachel E Powers, Joseph Marcus, Mary B Ford

**Affiliations:** San Antonio Uniformed Services Health Education Consortium, San Antonio, TX; Brooke Army Medical Center, San Antonio, TX; Brooke Army Medical Center, San Antonio, TX

## Abstract

**Background:**

While interferon-gamma release assays (IGRAs) are designed for detection of latent tuberculosis infection (LTBI), they are often used by inpatient providers. This study analyzed the use of inpatient IGRAs at a single-center to determine inpatient test utility.

**Figure:**
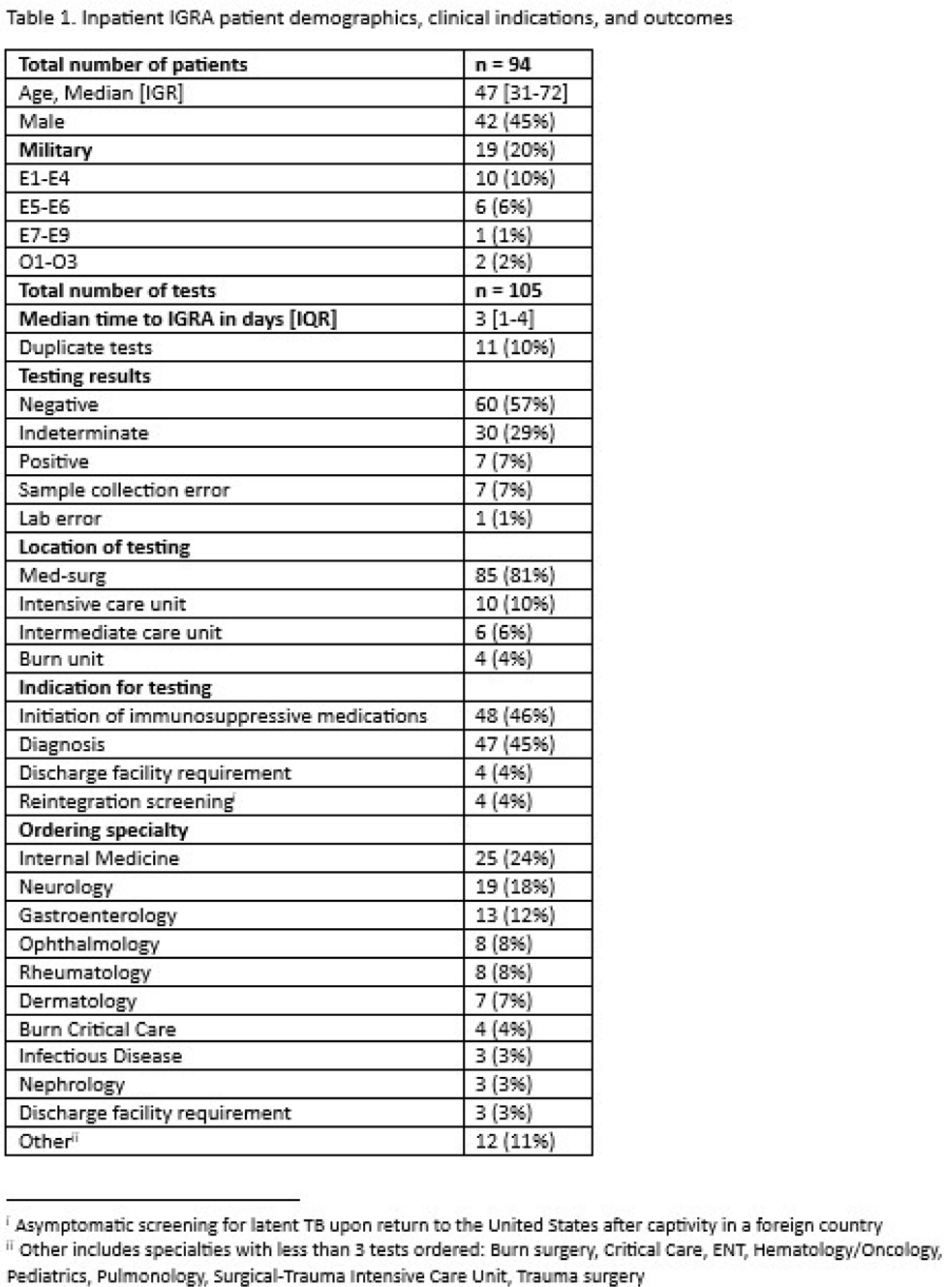

**Figure:**
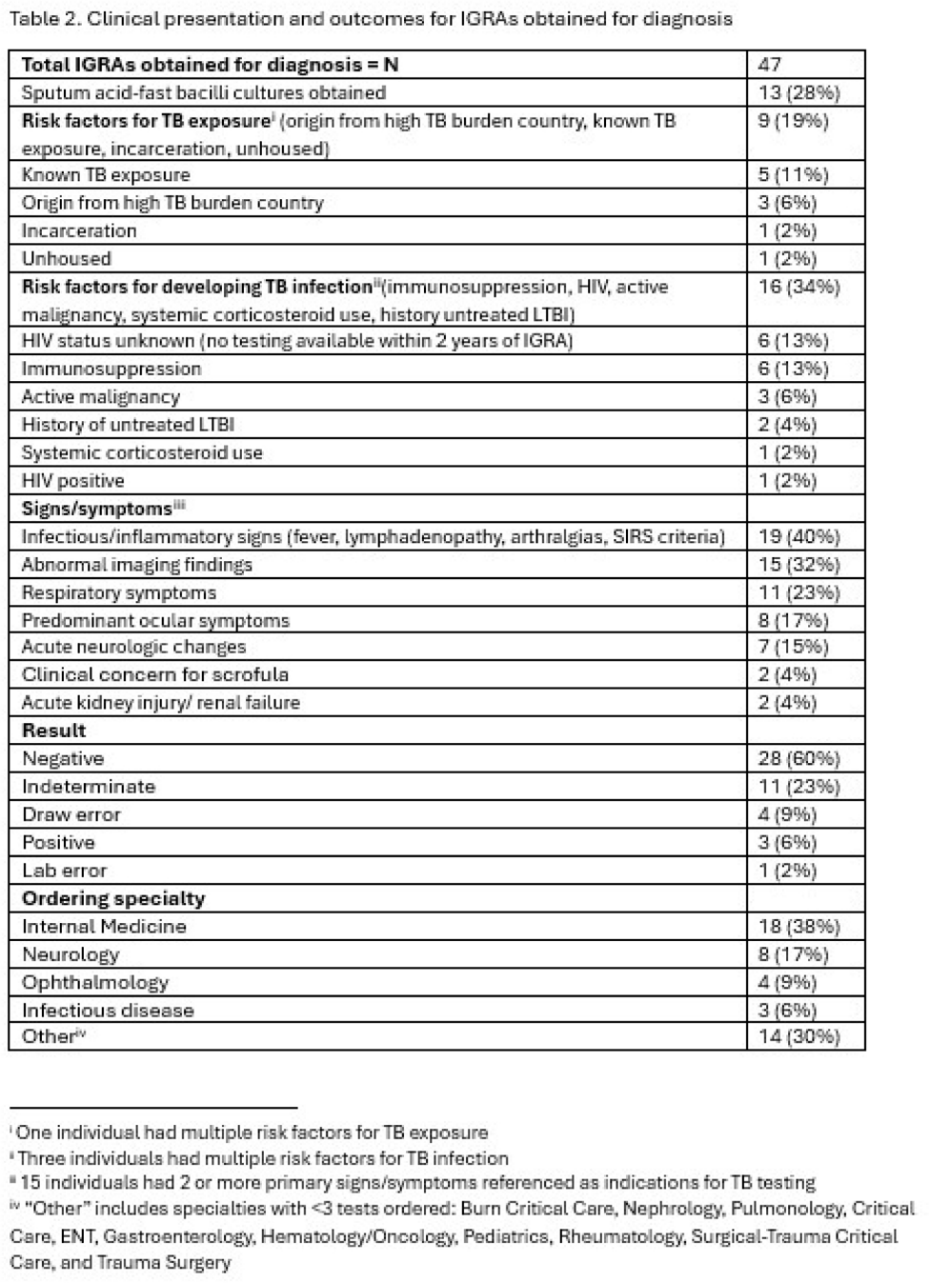

**Methods:**

A retrospective chart review was performed of all inpatient TB-QuantiFERON IGRAs for Brooke Army Medical Center from January 1, 2024 to December 31, 2024. Data were collected regarding patient demographics, ordering medical specialty, test result, tuberculosis (TB) infection risk factors, follow-up testing, and clinical outcomes.

**Results:**

Of the 3,731 IGRAs ordered during the study period at Joint Base San Antonio, 105 (2.8%) were obtained from inpatients, with 7 positive (7%) and 30 indeterminate (29%) results. Rates of indeterminate IGRAs were significantly higher for inpatients than outpatients (29% vs. 1%, p< 0.0001). Inpatient IGRAs occurred more commonly in females (55%) with a median age of 47 [IQR 31-72] and median day of hospitalization IGRA was obtained was 3 [IQR 1-4]. General internal medicine providers ordered the most IGRAs of any specialty (24%), followed by neurology (18%), and gastroenterology (12%). Indications for IGRAs included initiation of immunosuppressive medication (48 [46%]) and diagnosis of active tuberculosis (47 [45%]). Of the 30 indeterminate tests, 20 (67%) were from patients receiving immunosuppression at the time of the test, and only 3 led to a change in clinical management. In patients with concern for TB disease, IGRAs were paired with sputum acid fast bacilli cultures for only 13 (28%) patients.

**Conclusion:**

While inpatient IGRAs accounted for a minority of IGRAs collected, they account for a disproportionally high number of indeterminate results. In the inpatient setting when they were collected for concern for tuberculosis disease, sputum cultures were collected only in a minority of patients. When compared to traditional TB diagnostics, IGRAs are an appealing test due to rapidity of results, however this data suggests re-evaluation of their use in inpatients. Further elucidating clinical presentations and patient demographics which may lead to inpatient IGRAs may identify testing algorithms or incorrect assumptions about IGRAs which can inform future testing procedures.

**Disclosures:**

All Authors: No reported disclosures

